# How does it feel to be a problem? Patients’ experiences of self‐management support in New Zealand and Canada

**DOI:** 10.1111/hex.12823

**Published:** 2018-09-22

**Authors:** Nicolette F. Sheridan, Timothy W. Kenealy, Anita C. Fitzgerald, Kerry Kuluski, Annette Dunham, Ann M. McKillop, Allie Peckham, Ashlinder Gill

**Affiliations:** ^1^ Massey University Auckland New Zealand; ^2^ University of Auckland Auckland New Zealand; ^3^ Encompass Research Auckland New Zealand; ^4^ Lunenfeld‐Tanenbaum Research Institute Sinai Health System Institute of Health Policy, Management and Evaluation University of Toronto Toronto Ontario Canada; ^5^ Institute of Health Policy, Management and Evaluation University of Toronto Toronto Ontario Canada; ^6^ University of Toronto Toronto Ontario Canada

**Keywords:** ethnic minorities, long‐term conditions, patient preferences, patient‐clinician relationship, PRISMS taxonomy, self‐management support

## Abstract

**Background:**

The impact of long‐term conditions is the “healthcare equivalent to climate change.” People with long‐term conditions often feel they are a problem, a burden to themselves, their family and friends. Providers struggle to support patients to self‐manage. The Practical Reviews in Self‐Management Support (PRISMS) taxonomy lists *what* provider actions might support patient self‐management.

**Objective:**

To offer providers advice on *how* to support patient self‐management.

**Design:**

Semi‐structured interviews with 40 patient‐participants.

**Setting and participants:**

Three case studies of primary health‐care organizations in New Zealand and Canada serving diverse populations. Participants were older adults with long‐term conditions who needed support to live in the community.

**Main outcome measures:**

Qualitative description to classify patient narratives of self‐management support according to the PRISMS taxonomy with thematic analysis to explore *how* support was acceptable and effective.

**Results:**

Patients identified a relationship‐in‐action as the mechanism, the *how* by which providers supported them to self‐manage. When providers acted upon knowledge of patient lives and priorities, these patients were often willing to try activities or medications they had resisted in the past. Effective self‐management support saw PRISMS components delivered in patient‐specific combinations by individual providers or teams.

**Discussion and conclusions:**

Providers who establish relationships with patients can support them to self‐manage and improve health outcomes. Delivery of taxonomy components, in the absence of a relationship, is unlikely to be either acceptable or effective. Providers need to be aware that social determinants of health can constrain patients’ options to self‐manage.

## 1 INTRODUCTION

Health systems risk being overwhelmed by the significant impact of long‐term conditions—the “healthcare equivalent to climate change.”^1^ Ongoing illness is affecting a growing number of older people, especially those who are poor and belong to ethnic minorities. Many experience multiple concurrent conditions that require complex care with different treatments and involve a range of various health‐care providers.^2^ People with long‐term conditions often feel they are a problem, a burden to themselves, their family, friends and even health providers. Patients, and providers, often struggle to “control” long‐term conditions and “failed management” is repeatedly cast as a patient problem,^3^ even though providers can have deficits in knowledge and confidence, face time constraints and find care coordination a challenge.^4^ There is an urgent need to transform how community‐based primary health care supports people with long‐term conditions to self‐manage, take more control over their health and improve their health outcomes. Providers must be aware of patients’ needs and preferences^5^ to be effective in improving patients’ health, defined as the “ability to adapt and to self‐manage.”^6^

A recent systematic review of reviews summarized evidence on interventions that support people to self‐manage their long‐term conditions.^7^ In the process of their review, the authors constructed a taxonomy of provider activities to support patient self‐management (14 components, see Appendices 1 and 2). Pearce et al^8^ presented the rationale and development of the taxonomy, which they called PRISMS (from the overall project: Practical Reviews in Self‐Management Support), and tested it against an existing support manual for patients who had survived cancer. The authors clearly distinguished between “direct” patient support by providers, and “indirect” support that providers themselves might receive from the organizations in which they work. They explicitly excluded indirect support from the taxonomy.

We subsequently assessed whether we could identify each category of provider activity within narratives from patients with long‐term conditions.^9^ We argued that the patient is the ultimate arbiter of whether self‐management support has occurred and been acceptable and effective. We identified 11 of the 14 components in patient narratives and found evidence for the others in narratives from their health service providers.

This study extends our previous work on the PRISMS framework^9^ using data from a wider range of patients. Our objective was to offer providers advice, from patients, on *how* to effectively support self‐management (the PRISMS framework advises on *what* providers might do).

## 2 METHODS

### 2.1 Setting

Data were collected in 2015 by interviews with patients in three case studies that were part of a programme of investigation into implementing community‐based primary health‐care services in New Zealand and Canada. We did not specifically seek a country‐comparison, but sought patient populations that differed by age and gender, as well as ethnicity and culture, geography (urban and rural) and model of primary care delivery. The organization (case study) is described only to give context to patient‐provider interactions. The unit of analysis is the individual patient, not the provider or the organization. The overall study and the case studies are described in more detail elsewhere.^10^, ^11^

Based in northern New Zealand, case study one was a not‐for‐profit community trust that for more than 20 years has delivered services to a rural population of approximately 20 000 of whom 5000 are indigenous Māori. Services include primary medical and nursing care, public health, mobile nursing and programmes in schools and marae (traditional meeting places). Care is delivered in clinics, or in patients’ homes, by a small multidisciplinary team that includes a nurse practitioner, doctor, nurses and community health workers. The Trust provides Kaupapa Māori Services, which emphasize Māori culture and values.

Based in southern New Zealand, the second case study is a network that was extended following the 2011 Christchurch earthquake when services needed to be rebuilt and redesigned. Organized by the local district health board and primary health organizations the network serves a population of approximately 540 000 and includes urban and rural general practices, nurses, pharmacists, homecare providers and allied health professionals. Specific programmes and funding are dedicated to reducing hospital admissions, including programmes that “pull” patients from hospital to home for short‐term intensive services, home medication management programmes and care coordination.

Based in Ontario, Canada, the third case study was a longstanding not‐for‐profit community care organization serving a population of 6500 whose services included meals on wheels, day programmes, homemaking, supportive housing and a multidisciplinary primary health‐care team. The organization was originally designed for, and continues to predominantly serve, the Chinese migrant population. Additional interdisciplinary assessment and care including collaboration between primary care and community services is provided for patients with complex care needs.

### 2.2 Participants and interviews

Participants were all patients within the case studies, had two or more long‐term conditions, and lived in the community (ie, not residential care). We defined a long‐term condition as ongoing or recurring and which could have a significant impact on a person's life. The definition included disability and mental health conditions. The age for inclusion was 50 years or older to accommodate Māori who have poorer health outcomes and a higher burden of multi‐morbidity than non‐Māori of the same age, often compounded by relative poverty.^12^ Participants were selected for variation by ethnicity (primarily Māori, European, and Chinese) and gender.

Patient interviews have been described elsewhere.^11^ Briefly, discussions were guided by patients’ responses to validated questionnaires about patient perceptions of managing everyday activities;^13^ assessment of health services for chronic illness care;^14^ culturally‐mediated experiences of health^15^ and the impact of their material standard of living.^16^

The majority of patients volunteered in response to posters in primary care practices. Nine patients were identified as potentially eligible to take part in the study by a nurse or care manager who asked their permission to pass contact details to the research team. Participants were interviewed at a place of their choice and were able to bring a family member or support person. All participants gave written consent to their interview being recorded. Six researchers, male and female, one of Māori descent (case 1) and one of Asian descent (case 3), conducted the interviews which lasted from 45 to 90 minutes. All New Zealand participants were interviewed in English, while six of the Canadian participants (case 3) were interviewed with an interpreter (five in Cantonese and one in Mandarin). Participants were known to providers, but not to the researchers. Digital audio filenames were coded to ensure anonymity after interviews. Interviews were transcribed verbatim omitting personal names; case 3 interviews were translated from Cantonese to English before transcription. In New Zealand, ethical approval was given by the University of Auckland Human Participants Ethics Committee (reference 013071) and in Canada by the University of Toronto Ethics Review Board (reference 128263).

### 2.3 Analysis

The PRISMS taxonomy—a summary of extensive literature on provider self‐management support to patients—offered a framework for analysis of patient data in relation to their own self‐management experiences. Thus transcripts were read for evidence of delivery of PRISMS components as experienced by patients (deductive coding), and we collated the data under codes relating to provider activities in the PRISMS framework. An inductive thematic analysis sought insight into how self‐management support was provided, what made it effective and acceptable to patients, and what they wanted, but did not get. This coding and analysis resulted in our grouping PRISMS components for reporting, because patients’ narratives described combined components. The coding was undertaken by AF and verified by NS and TK. Quotes were confirmed by consensus of all authors to illustrate issues patients perceived as most important.

## 3 RESULTS

Forty patients were interviewed across the three case studies (see Table 1). They were aged between 50 and 94 years and 25 were women. The majority of participants in case one were Māori, in case two were New Zealand European, and in case three were Canadian Chinese.

**Table 1:** Describing patients interviewed (n = 40)

Case	Ethnicity		Gender		Age	
Case 1	Māori	13	Male	9	50‐64	6
	NZ European	2	Female	6	65‐74	5
					≥75	4
Case 2	NZ European	9	Male	3	50‐64	0
	Other European	1	Female	7	65‐74	2
					≥75	8
Case 3	Chinese	13	Male	3	50‐64	1
	Canadian European	1	Female	12	65‐74	2
	Guyanese	1			≥75	12

PRISMS component 1 (provision of information to explain the patient's long‐term condition) was the most frequently identified. Provision of easy access to support or advice when needed (component 8), lifestyle advice or support (component 14), and practical support with adherence (component 6) also featured frequently in patients’ narratives. Components not frequently identified included provision of, or agreement on, specific clinical action plans and/or rescue medication (component 3), information about available resources (component 2) and monitoring of condition with feedback (component 5). Components 9, 10 and 11 were not identified; these describe training or rehearsal to communicate with health‐care professionals, for everyday activities and for practical self‐management strategies, respectively. In general, and for each component, we found as many examples of good as of poor self‐management support, which taken together strongly confirmed the same points.

When interpreting the data, we were struck by a sole emergent unifying theme: relationships as the primary and pervasive method to enable providers to deliver effective self‐management support and that patient self‐management is intermediary between relationships and improved patient outcomes.

While we document multiple examples of *what* providers did (Appendix 1), we focus here on *how* patients wanted to be supported because this offers actionable guidance to providers. First, we expand on the importance of relationships, and how these are manifest, followed by a series of provider actions and patient experiences in relation to specific PRISMS components.

### 3.1 Relationships—*how* to support self‐management

Patients perceived self‐management support as effective when they had a relationship with their nurse, nurse practitioner, doctor or community worker, no matter what component was delivered. When patients did not have a relationship with such a person they were less likely to disclose information that could prompt providers to recognize opportunities for appropriate self‐management support. One hallmark of a good relationship was that the provider was able to elicit and receive information from patients that reflected their needs.

I always feel [the GP] listens. And, if I get [the nurse], she listens. And, through their questioning, they are able to pinpoint what it is I'm actually there for and… ‘Let's get this seen to…’ I come out of it feeling happy, because somebody has actually listened to me and… mapped a pathway for us to follow to get down to what actually is going on. (case 1, female, 62 years)

The information shared in two‐way listening led patients to want to engage.

We always put our ideas down, and then she tells us what her ideas are, so it's open, two‐ways… we feel a lot more freer to speak. You know? (case 1, male, 70 years)

An older Chinese woman explained that she felt rejected and a burden to her son and daughter‐in‐law with whom she lived, but felt scared and alone when they went on holiday. She said, “I think I am more happy to see you people [care team] than seeing my own family” (case 3, female, 75 years). Her GP organized for her to join a Tai Chi class he ran so that she could meet other Chinese people.

He [my doctor] said, ‘You have depression. You better come here. You will be more happy. You'll see more people. You can talk to each other.’ The family doctor always does the exercise things free of charge…He teaches us the Tai chi. (case 3, female, 75 years)

When providers recalled, without having to ask each time, important aspects of patients’ lives, such as who they lived with and who they had lost, patients interpreted this as evidence their provider cared. Relationships were both supported by, and in turn reinforced by, continuity of care with individual providers.

… every time you go in there, it's very personalized; [Nurse Practitioner] doesn't have to look up your name on anything, nor does [GP] like that. Both of them, I think both of them are the two people that are central to my health. (case 1, female, 62 years)

The same point is made in the following negative example.

But, the thing is, I don't see Dr X much at all. Every time I ring up for an appointment with him, he is either too busy or I have to see one of the doctors, and I'm not there for one of the other doctors; I'm a client there for Dr X, not the other doctors. (case 1, female, 84 years)

Continuity of individual provider contributed to feeling safe with a person who knew them was monitoring their current health status and knew their often complex medical history.

Relationships naturally led to negotiating patients’ preferred involvement in decision making and their preferred level of autonomy, which varied widely. Older Māori patients, enrolled with the Māori provider organization, expected respect as a cultural norm. Most patients wanted to actively contribute to decisions.

Some, particularly Chinese migrant participants, responded “I just follow the doctor's instruction” and did not presume to have additional ideas about their treatment, or to challenge the “experts” who were providing their health care. “I don't have the knowledge or expertise to make part of the decision. I don't feel I have the knowledge” (case 3, female, 75 years).

The most frequently noted issues of practical importance were providers’ knowledge of patients’ financial hardship, and acting on that knowledge. Affordability of primary health‐care services was important for all older patients in our case studies, most of whom were living on a pension, or had limited retirement savings. New Zealand patients spoke of general practitioners and nurses providing dressings at no cost. In one case study, clinicians aided access to low‐cost food outlets.

… they've got a lot of places that you can go to, they've got a $20 box of veges down at Vege Twins and you get potatoes, you get carrots… so that's what I get every week, and you get your fruit in it. And you also get a tray of eggs. (case 1, female, 59 years)

Pre‐emptive contacts and support were further evidence of relationships and care that were particularly valued by patients.

I think they go the extra step… They'll call, even after your [operation], they call you to see how you are, you know? And that's really special. It makes you feel special… they've never not called… we've gone two or three weeks, maybe, tops, without seeing them, she'll always ring up to make sure, ‘Is everything alright?’. Yeah… they're interested in your whole well‐being. (case 1, female, 62 years)

Relationships and self‐management support enabled some patients to extend that support to family and friends; a useful outcome marker of effective provider support.

So, I've talked to all the kids, and we have mayonnaise but we'll have aioli now, we'll have that. We thought that Edam cheese was the best for us, but we've found out that cheddar cheese is better than Edam…there's a whole lot of things that we have found out, and my daughter has gone from 110kgs down to 70kgs. (case 1, female, 59 years)

### 3.2 PRISMS component‐specific *how*

Eleven of the 14 components of the PRISM taxonomy were identified in patients’ narratives. We found that each component was connected to other components. Providers naturally grouped activities, revealing *how* they operationalized self‐management support. We found, in patient narratives, combined components that we have summarized in four groups to simplify reporting. These are information giving (components 1, 2, 14); clinical planning, review and feedback (components 3, 4, 5, 12); service access, coordination and social support (components 7, 8, 13); and practical help with medication (component 6).

#### 3.2.1 Information giving (components 1, 2, 14)

Providing information was seen as the most basic requirement of self‐management support. To be effective, some patients needed the clinician to take more time explaining, perhaps repeating it several times before they properly understood.

I don't know if I'd be able to go back to a regular doctor…. I'll just get hoha (irritated) and everything will just go out the window again… they haven't got the time to explain what your medication's for, and why. They had me on some really high dosage of medication, and even [clinician] couldn't understand what they were, and why they were so high. (case 1, female 59 years)

When providers did take time, patients felt more confident to self‐manage.

Yes, it took several conversations with [the nurse] for me to realize that it's not a bad thing to take an anti‐inflammatory. (case 1, female, 62 years)

I think that's why I liked going there, because they'll take the time to go over and over and over again with you about what it is that's taking place and what your options are. (case 1, female, 62 years)

Speaking the same language, “My family doctor can speak Cantonese… I trust *him a lot. It's been a few decades since I started seeing him”* (case 3, female, 87 years) made communication easier. This experience was in direct contrast to that of another participant who simply did not understand at all because of a lack of basic translation services.

But I feel like all the healthcare providers in the hospital, they were very polite to me. But in terms of the medication or mental care, I don't understand either. And nobody explained to me thoroughly what's going on. So what I did is after that, I printed the report, printed it out, and then I used a translation online to have it translated to see what's going on. (case 3, male, 65 years)

Patients in our case studies, on the whole, appeared to be receiving the financial assistance they were entitled to. Practical assistance by providers to access local services or to identify food banks or low‐cost community markets appeared to strengthen the underlying relationship because participants saw this as the provider understanding them in their current circumstances.

Information on lifestyle factors was targeted. One woman said, “I am taking more care to look at what's on the back of the tin I pick up, so I'm looking for low salts, low sugars…” (case 1, female, 62 years). She said written information about a healthy diet had not had an impact, but having the dietician visit the supermarket with her and show her how to read the labels on different food items gave her the confidence to make healthier choices. The dietician elicited the patient's food preferences and what she knew, before providing information to fill gaps in knowledge and understanding. In addition, how the messages were delivered was seen as acceptable:

…the dietician was very, very helpful…friendly…. in the way she offered up in the information, there is a bit of joking, character and all, she made me feel very comfortable. (case 1, female, 62 years)

#### 3.2.2 Clinical planning, review and feedback (components 3, 4, 5, 12)

Participants expected to agree on a clinical action plan they understood, relevant to any stage of their life or illness. An older woman with asthma said “They give me some paper but I am not able to read it” (case 3, female, aged 83 years). Another woman commented “The doctor said there's not much you can do because of the age. Like its general deterioration of the body” (case 3, female, 81 years). For several patients nearing the end of their life, we could not identify conversations or plans that addressed patients concerns about, for example, how pain might be managed. A lack of engagement could leave patients with unaddressed feelings of hopelessness, only compounding their anguish.

What value do I have as an old person? I'm 80‐some years old. What value do I have? I don't have much value. I have to leave it to the Lord Jesus to arrange when I will leave this earth. (case 3, female, 87 years)

Regular clinical reviews allowed clinicians the opportunity to assess psychological well‐being and adjust care in response. “I feel like I can't make it as a person. My whole body. Like at my age, I'm taking so much medication, I might as well just pass away” (case 3, female, 81 years). An awareness of the patient's psychological state was important. Setting goals, or recording events, when a future couldn't be envisaged, was distressing. “No, I don't want to think about myself [and use a log book], because I get… I'm sure I'm dying” (case 2, female, 85 years).

Regular reviews were hugely valued, as in the following example where the clinician regularly visited people at home, at agreed times.

… this girl (Nurse Practitioner), she's just come on board now, she is wonderful. We've had other people… they haven't lasted long, and their visit haven't been – what do you call it – continuous. You'll see them one fortnight; you won't see them for another few months. (case 1, female, 66 years)

#### 3.2.3 Service access and coordination and social support (components 7, 8, 13)

Multiple examples revealed that people were left feeling disempowered and frustrated after poor service coordination, which often occurred over many weeks. For example, the direct action of providing equipment was not enough. The timing of equipment provided, and the follow‐up once equipment had been provided were identified as important dimensions of care. A frail elderly woman needed a hospital bed to remain living independently. There was confusion when the bed was delivered unexpectedly, the mattress was not correct, and the contractor did not install the bed.

Well, I actually rang after the bed arrived, I'm sitting there…I was getting all uptight and that, and I rang occupational therapy [at the hospital] and complained about it, and they said, ‘well, you're lucky, lucky you've got a bed, aren't you?!’, and that's all I got from them. And I'm thinking, yeah, well, I live alone, bed's in the garage, what do I do about it? (case 2, female, 79 years)

She explained she could not move the bed herself physically.

They were supposed to take my old bed and store it somewhere and put the hospital bed in my room. And they didn't do it.

Eventually, “…my son‐in‐law… he pulled the headboard and the other end, disconnected the power, where the power goes and everything like that, he had it up in about 3 or 4 minutes.” This woman, like others, was described by the interviewer as lacking trust in her provider after feeling let down.

In contrast, some primary care providers helped patients to access hospital specialist services and to access social welfare services. The latter extended to identifying benefits available to patients and also often accompanying them to the government welfare office.

Yeah, they give you an accommodation supplement, I get $80, and this place is $370 a week, so it's not much, but it does help, anything helps…. I only go there as a last resort… you feel so terrible that you've got to go there and ask for this and ask for that. (case 1, male, 50 years)

[After I was discharged] They told me all about [pause] the social worker told me about people that can come and help and all that sort of thing. (case 2, female, 78 years)

#### 3.2.4 Practical help around medication (component 6)

Medication adherence could be importantly limited by financial and physical access to medications. Patients in this study did not need (or receive) telephone calls or other reminders to refill prescription medicines from providers in any of the case studies. Patients in New Zealand cases appeared mostly to have good health literacy, explaining how they took medications to allow maximum activity and reduce side‐effects. Some patients in the Canadian case described the challenges of language and of literacy.

We don't speak good English…. we don't understand many medications…. sometimes, for example, we are a little busy and we tend to delay it. And then by delaying, it will cause big troubles. (case 3, male, 65 years)

The impact of cost was described by one man who said,

I think that's the only thing that gives us a bit of a hard time, is the cost of the prescription…. the wife's always saying, ‘Oh, we've got to save up for that,’ because it's $5 a prescription at the chemist… it turns out to be about five or six prescriptions, you now…. so those are the costs, and sometimes it adds up quite a bit, $5 a prescription. (case 1, male, 73 years)

One pharmacist routinely supported patients to access medications, with one person commenting “He [the pharmacist] lets me pay it off, otherwise I wouldn't be able to get any of my medication” (case 1, male, 50 years) and another, “He's a really good, good guy [the pharmacist]. I don't always have the money to get my pills, he'll let me pay it off, he's really good” (case 1, male, 50 years). Others planned ahead, “I just take it in [prescription] and then I'll go pick it up on payday” (case 1, male, 68 years).

Several older patients relied on family members or friends for transport to collect medication. There were also repeated examples of primary care providers delivering medication to older patients whose physical mobility was limited, or who lived in rural places without transport.

He [pharmacist] knows I can't go without my medication. And sometimes if I'm stuck at dialysis, because my wife doesn't drive, he'll drop my pills down here or drop her pills here. They just do the extra distance for you, it's really good. (case 1, male, 50 years)

One 83‐year‐old woman explained that because of her arthritis she was unable to open the blister pack containing her medication. She explained, “…the people from [case study three], every morning they come here and take the medication from my blister packing and help me with my medication” (case 3, female, 83 years).

## 4 DISCUSSION

This study sought to identify, from the narratives of a diverse group of patients, *how* providers might effectively support self‐management (the PRISMS framework classifies *what* providers might do). Patients valued relationships with providers that were characterized by listening, caring and shared decision making; were supported by continuity of care; and where providers knew the context of their lives and acted upon this knowledge. Gawande argues that for those with chronic conditions trust underlies incremental “steady, intimate care”^17^ that can help patients. Patients actively engaged with and trusted providers whose actions directly addressed their clinical and non‐clinical needs. Patients who experienced a relationship of trust often described to providers their everyday social, physical, emotional and financial challenges, and were often willing to try out activities or medications they had resisted in the past. The central desire of patients for a relationship with providers is well established in the literature.^5^, ^18^, ^19^, ^20^ However, what we propose here goes further to suggest that a relationship‐in‐action is the central *how*, the mechanism by which providers can support patients’ ability to “adapt and self‐manage.”^6^

Health care provided within clinician‐patient relationships includes emotional care (trust, empathy, acceptance and warmth) and cognitive care (information, managing expectations and education) and has been linked to improved patient outcomes.^21^ This may be because relationships determine the quality and completeness of information that is elicited and understood^22^ and better information given and received in turn enhances health outcomes.^23^ We are also suggesting that patient self‐management is the intermediary step between relationships and improved health outcomes.

We have previously asserted that the onus to facilitate communication and relationships with patients lies with health providers. This is essential when patients experience powerlessness as a result of the compounding jeopardy from chronic conditions, poverty, minority status and age.^5^ Providers always cite lack of time as a primary limitation on their ability to offer ideal care, including a lack of time to convey information and explanation (or translation) in a way that patients understand.^24^ Some authors suggest it is not the actual time available that matters, but what is done within that time,^23^ and note that actual time can be perceived differently by clinicians and patients.^25^ Many of the patient narratives suggest that providers who invest time early in a relationship can establish a level of trust and self‐disclosure that later supports more efficient clinical care. We noted that nurses and community health workers often contributed knowledge about an individual patient that enabled a doctor to respond more effectively, despite often short consultations.

If a relationship is central to effective service provision, we need to find ways to operationalize it. We think it is useful for providers and their organizations to explicitly adopt an agreed frame of reference regarding the provider‐patient relationship. This should frame patients and providers as active partners in managing conditions, in which the patient is an expert, particularly in their own preferences, priorities, resources and values, while acknowledging that patients have the power to veto any recommendation made by a provider.^26^ Many such models exist, and, unsurprisingly, overlap extensively^27^ even though their language varies, and some emphasize provider attitudes and values while others emphasize provider actions. Examples of the former include an empowerment approach,^27^, ^28^ a person (or client) centred approach,^29^ patient‐centred medicine^30^ and person‐centred medicine.^31^ Examples of the latter include Motivational Interviewing,^32^ Brief Opportunistic Interactions^33^ and “health coaching.”^34^ What these models have in common is their use of well‐structured open questions that help to “elicit” patient knowledge so that the provider can “provide” what is missing following an “elicit‐provide‐elicit‐provide” pattern.^35^ Example questions are given in Table 2. Each of these models requires specific training for providers.

**Table 2:** Typical health provider questions, based on Brief Opportunistic Interactions,^33^ to elicit patient information, priorities and intentions

Tell me what you already know about (condition) and what that means for you?
What are the things you enjoy most about (an activity eg going to the gym)?
What do you dislike most about taking medication?
What have you noticed most since changing the way you (do an activity, eg exercise)?
What is the main thing that triggers your sadness?
What do you enjoy about (risk behaviour eg smoking?) And what's not so great?
If you could change one thing in your life at the moment what would that be?
How important is it to you to change (specific behaviour or activity)?
How confident do you feel that you can change (specific behaviour or activity)?
What could be getting in the way of changing (specific behaviour or activity)?
How do you feel about making a plan together next time you come?

What these models also have in common is that they seek to build a relationship for a health‐enhancing purpose, that is they lead to provider action. We found that providers combined PRISM taxonomy categories. This was a practical way to organize care to meet patients’ specific needs. We discuss provider actions under four groups: information giving; clinical planning, review and feedback; service access, coordination and social support; and practical help with medication.

Information giving related to condition diagnosis, prognosis, management, resources and lifestyle advice. It was often partial and often one‐directional, in which case it could be paternalistic. It was perceived as acceptable and useful only if delivered in the context of knowledge about the person, which comes only through two‐way dialogue. Clinical planning, review and feedback included agreeing on an action plan that would enable a patient to self‐assess and adapt their own actions, if needed. This could be achieved only by working with the patient, checking their understanding, allowing people the autonomy to select strategies that are practicable while meeting their personal needs and priorities. We assigned practical help with medication to its own group given its importance in management of long‐term conditions. Medication has great potential for benefit, or for harm. It is frequently used improperly and frequently not taken regularly by the patient even when benefit is likely. In this context, the World Health Organization has described improving medication adherence as the biggest single changeable factor to improving outcomes for patient with long‐term conditions, and probably in reducing health inequities for groups who are socially disadvantaged.^36^ Patients repeatedly commented with gratitude when providers improved their access to medications by helping with cost, transport or physical access such as blister packs. Coordination of services, practical help and facilitating access to services all featured prominently in patient narratives. In the PRISMS taxonomy, this category included providers linking patients to social support. While there were good examples of one or more taxonomy components being delivered, we also heard examples of poor coordination, failure to help patients access the help they needed, and patients having to call on others within their social support network to solve gaps in service provision. Patients talked of incomplete delivery of single components, disconnect between components when several were delivered, and not all patients with the same need receiving the same self‐management support (as required by equity). What was offered to patients seemed provider‐dependent, and perhaps time‐ and chance‐dependent, rather than patient‐dependent. The overall impression was of reactive and partial response to current patient need for self‐management support, with limited coordination to ensure complete delivery. There was no evidence of long‐term planning for long‐term conditions.

While these groupings reflect the narratives of patients’ in these cases, other patients narratives might have prompted other groupings. Nevertheless, we think that any combination of activities reflect the fact that the clinical and personal needs of any one patient are rarely met by just one provider action, and for that reason, self‐management support is typically delivered in a way that meets several taxonomy categories at the same time. The taxonomy, as a list, is not sufficient by itself to define *what* must be done to support self‐management; and it has nothing to say about *how* self‐management support needs to be delivered.

One limitation of much of the self‐management literature is that most participants are highly self‐selected as interested, motivated and able to develop self‐management skills.^37^ A recent Cochrane review confirmed that a large number of participants in studies of self‐management education felt their condition to be relatively stable and their health to be reasonably good.^38^ A strength of this study was the real‐life context—patients in two jurisdictions were interviewed in their own homes or community health facilities, and we were able to both see and hear how the PRISM framework applied to them managing long‐term conditions in everyday settings. The interviews were not specifically targeted at identifying components of the PRISM framework, which may be considered a limitation. However, interviews explicitly enquired about self‐management support and the relevance of this in the broader context of people's lives. A final limitation is that individual patient narratives were analysed in terms of interactions with providers who offer “direct” support. We acknowledge that organizations can offer “indirect” support to providers who work in them; however, the PRISMS categories were constructed explicitly excluding the effect of organization on provider self‐management support. Indirect support has been outside the scope of our analysis, but is an important area for future inquiry.

## 5 CONCLUSION

The unavoidable necessity for patients to self‐manage, and the central desire of patients to have a relationship with providers, are well established in the literature. What is new is that relationships are not usually characterized as the primary and pervasive method to enable providers to deliver effective self‐management support and that patient self‐management is intermediary between relationships and improved patient outcomes. In our cases, and others reported in the literature, there is little attention to either planned delivery of self‐management components (as in the PRISMS taxonomy) or to fostering relationships for the purpose of self‐management support. We suggest that strong patient‐provider relationships built on trust and mutual respect can strengthen effective self‐management support, and vice‐versa in a virtuous circle. We also suggest that, by routine attention to self‐management support for patients with long‐term conditions, providers could reasonable expect to save time while increasing effectiveness, but this is for future investigation.

## CONFLICT OF INTEREST

All authors declare they have no conflict of interests.

## APPENDIX 1 PRISM Taxonomy components (numbered 1‐14) and descriptions, adapted from Pearce et al^8^

### 1.1

**Table:**

1	*Information about condition(s) and/or management* Information about symptoms, condition(s) including prognosis, and interventions (eg medication, emotional and psychosocial) to people with LTCs and their families
2	*Information about available resources* Information about financial benefits, social, community or peer support, and charitable organizations or health support organizations for people with LTCs and their families
3	*Provision of/agreement on specific clinical action plans and/or rescue medication* Written instructions prepared with a health professional to enable people with LTCs and their families to take an approach tailored to the person, condition(s), and severity. This includes taking medication, and recognizing symptom deterioration and taking action
4	*Regular clinical review* A regular scheduled review with a health professional about symptom and condition management, and the support needed to self‐manage
5	*Monitoring of condition with feedback* The person with the LTCs and their family, or health professional, monitor symptoms, behaviours or objective measures related to the LTCs. The person has access to their results. Interpretation, decisions and actions may be supported by a health professional
6	*Practical support with adherence (medication or behavioural)* Practical help offered to support adherence to medication or change at‐risk behaviour
7	*Provision of equipment* Provision of equipment to enable self‐monitoring and/or self‐management of the LTC
8	*Provision of easy access to advice or support when needed* Timely access to health services delivered flexibly when presenting with an urgent or a non‐urgent
9	*Training/rehearsal to communicate with health‐care professionals* Teaching people with LTCs to develop communication skills to improve relationships, better communicate their needs, and enhance shared decision making with health‐care professionals. Supporting people to practise skills they have been taught
10	*Training/rehearsal for everyday activities* Teaching people with LTCs to develop skills that support everyday activities. Supporting people to practice skills they have been taught
11	*Training/rehearsal for practical self‐management activities* Teaching people with LTCs to develop specific practical skills that will enable them to manage their LTC. Supporting people to practise the skills they have been taught
12	*Training/rehearsal for psychological strategies* Teaching people with LTCs skills to use psychological strategies to better manage the consequences of LTCs. Supporting people to practice the skills they have been taught, such as, problem‐solving, relaxation techniques, re‐framing, distraction, cognitive restructuring, goal setting and action planning
13	*Social support* Facilitation of social support to extend care. Includes peer support, social or community networks, charitable organizations and health support organizations
14	*Lifestyle advice and support* Practical general advice and support about health and lifestyle (eg physical activity, smoking cessation, diet and alcohol consumption). Not psychological strategies (see 12)

Note

LTC = long‐term condition; components 9‐11 not found in our patient data.)

## APPENDIX 2 Examples of *what* providers did to support patient self‐management

### 2.1

**Table:**

*Component 1. Information about the condition(s) and/or its management*
“I had a heart attack, after I buried my husband. So this time last year, I just wasn't handling it… I just didn't understand what was actually wrong with me, because I thought there was nothing wrong with me… I was always short of breath.” (case 1, female, 59 years). This woman assumed her problems were linked to her husband's death, but had a chronic condition. Her provider explained her condition, the medication she needed and established a treatment plan with weekly contact so that she felt supported “Yeah, I suppose [information] does matter to me… ‘cause it's my life that's being affected, isn't it, really.” (case 2, female, over 74 years)
*Component 2. Information about available resources*
“That lady… suggested I get a smaller walker…. I don't know who brought it to me, but when I got it, I said “I don't want a walker” she said ‘keep it because you might need it’. Well, see, eventually I did.” (case 2, female, 94 years) One man explained that despite having information, he worried for days before contacting social agencies. “Yep, I've had needs assessments and I'm always dealing with them [government welfare service]. It's not one of my favourite things…. because you feel so terrible that you've got to go there and ask for this and ask for that.” (case 1, male, 50 years)
*Component 3. Provision of/agreement on specific clinical action plan and/or rescue medication*
“Yep, we've got a plan, it's called a disaster plan, that we have to go through, and we update it every so many months, just so that if something happens they can contact us or we can contact them, or we know what to do.” (case 1, male, 50 years) “Yeah they asked me about a plan with my lungs when I went in. And they came and done a plan for me…. Yeah, they gave me a copy of it and everything.” (case 2, male, 79 years)
*Component 4. Regular clinical review*
“The quality [of care] is very, very good. I think it is anyway. Like, I'll put it this way, because she [nurse practitioner] comes out once a fortnight… they come out here and they service the area… to see the other people, the people that need it” (case 1, male, 82 years). The Nurse Practitioner undertook clinical reviews of people with long‐term conditions living rurally “For my family doctor, I went to see him on monthly basis. Usually I just attended an appointment. And before I left, I would talk to the nurse or the secretary there and arrange for my next visit.” (case 3, female, 75 years)
*Component 5. Monitoring of condition with feedback*
“One [goal] is to lose weight, they've been wanting me to lose weight. So I have to start losing weight for my own good, and I know it's for my own good.” (case 1, female, 58 years). This woman is monitored by the practice nurse who provides routine feedback and support “Yes she [practice nurse] always says I have to have to do my blood tests every week for Warfarin, just rings me and fill all the forms.” (case 2, female, 94 years). Close monitoring and feedback ensured care could be safe and responsive to this woman's needs
*Component 6. Practical support with adherence (medication or behavioural)*
The most basic requirement to support medication adherence, in particular, is to ensure transport and cost barriers do not stop patients obtaining medication. “…they deliver it [medication] because they know that I can't go.” (case 3, female, 80 years) “He's a really good, good guy [the pharmacist]. I don't always have the money to get my pills, he'll let me pay it off, he's really good.” (case 1, male, 50 years)
*Component 7. Provision of equipment*
[Nurse practitioner] said, ‘Have you got crutches?’ I said, ‘No.’ ‘Have you got a wheelchair?’ ‘No.’ And she organized all of that. And they were there that day. (case 1, female, 50‐64 years) “I've got this really hard mattress, and the occupational therapist said ‘oh, it's probably because of your body weight … because they're all memory foam, and so she said, ‘oh, I'll order another one for you, a better one’, so I didn't hear from her for about 6‐8 weeks” (case 2, female, over 74 years)
*Component 8. Provision of easy access to advice or support when needed*
“Actually, I know if anything… if I needed anything, needed to know anything, all I need to do is to ring [nurse practitioner]… if she doesn't see me straightaway, she'll make room for me next day.” (case 1, male, 73 years) “That's an awful thing for me to say but you have to ring and ring and ring… I rang yesterday and I actually lost all the [battery] power in the phone.” (case 2, female, 85 years). This negative example illustrates the basic need to be able to contact a front‐line health provider Good primary care is insufficient if more specialized help is unavailable when needed. “They all treat me nice… if I have to be referred to a doctor who is more specialized, you have to wait a long time. This is almost six months. I mean I will be dead by then” (case 3, female, 84 years)
*Component 12. Training/rehearsal for psychological strategies*
In this negative example, the patient felt dismissed due to his age rather than helped to develop personal goals relevant to his/her age and health. “Yeah, they all [my friends] went on top of one another… My doctor's response was “Well, older people die!” I said “Yeah, but not all at bloody once!” Yeah… older people die, as if I didn't know it…” (case 2, male, 79 years). “Earlier on, I still thought I could [set goals]. But now, you know, even the doctors have told me that there's not much that they can do. It's just mainly up to me.” (case 3, female, 84 years). This woman felt that opportunity was lost to set goals relevant to their life and illness.
*Component 13. Social support*
“I can tell other people, I've told a friend of mine that's got to go in for triple bypass… I said to him, ‘It's because you're smoking. Because you're boozing, because you're doing this, I know because I've been there.’ And it's been three months for him now… and I said, ‘See, I told you, it works’.” (case 1, female, 59 years). Patients supported each other, extending effective self‐management support beyond the health provider/patient dyad “So they will push my wheelchair to join them… so there's something like a get‐together and then we each have a meal together, whenever the care attending person tells me that there's some activities going on and they push me there, I will join them.” (case 3, female, 80 years)
*Component 14. Lifestyle advice and support*
“And she [nurse] rings me up if things are not right, she lets me know all about it. She's really good actually. She said to me last time “You're 50.” She said, “The time before you're 49, this time you're 50, and if you don't start and cut things down a bit in sugar and that, don't have any more beer…” I said, “Go to buggery, I'm still gonna have more beer”… I said “I might cut a little bit of sugar out” (case 2, male, 79 years) “Well, they are always bringing it up [eating and exercise]… I tell her that I go every Monday… go to Cardiac, to sit in the chair and do these little exercises. (case 1, male, 73 years) “She [health provider] told me to walk up my hill. ‘Oh, it's only half an hour, just walk up, around and around.’ And it did make a big difference.” (case 1, female, 59 years)
